# Soluble receptor for advanced glycation end products in COPD: relationship with emphysema and chronic cor pulmonale: a case-control study

**DOI:** 10.1186/1465-9921-12-37

**Published:** 2011-03-30

**Authors:** Massimo Miniati, Simonetta Monti, Giuseppina Basta, Franca Cocci, Edo Fornai, Matteo Bottai

**Affiliations:** 1 Department of Medical and Surgical Critical Care, University of Florence, 50134 Florence, Italy; 2 Institute of Clinical Physiology, National Research Council, 56124 Pisa, Italy; 3 Tuscany Foundation "Gabriele Monasterio", 56124 Pisa, Italy; 4 Unit of Biostatistics, Department of Environmental Medicine, Karolinska Institutet, 70177 Stockholm, Sweden; 5 Division of Biostatistics, Arnold School of Public Health, University of South Carolina, Columbia, 29208 SC, USA

## Abstract

**Background:**

The receptor for advanced glycation end products (RAGE) is a multiligand signal transduction receptor that can initiate and perpetuate inflammation. Its soluble isoform (sRAGE) acts as a decoy receptor for RAGE ligands, and is thought to afford protection against inflammation. With the present study, we aimed at determining whether circulating sRAGE is correlated with emphysema and chronic cor pulmonale in chronic obstructive pulmonary disease (COPD).

**Methods:**

In 200 COPD patients and 201 age- and sex-matched controls, we measured lung function by spirometry, and sRAGE by ELISA method. We also measured the plasma levels of two RAGE ligands, N-epsilon-carboxymethyl lysine and S100A12, by ELISA method. In the COPD patients, we assessed the prevalence and severity of emphysema by computed tomography (CT), and the prevalence of chronic cor pulmonale by echocardiography. Multiple quantile regression was used to assess the effects of emphysema, chronic cor pulmonale, smoking history, and comorbid conditions on the three quartiles of sRAGE.

**Results:**

sRAGE was significantly lower (p = 0.007) in COPD patients (median 652 pg/mL, interquartile range 484 to 1076 pg/mL) than in controls (median 869 pg/mL, interquartile range 601 to 1240 pg/mL), and was correlated with the severity of emphysema (p < 0.001), the lower the level of sRAGE the greater the degree of emphysema on CT. The relationship remained statistically significant after adjusting for smoking history and comorbid conditions. In addition, sRAGE was significantly lower in COPD patients with chronic cor pulmonale than in those without (p = 0.002). Such difference remained statistically significant after adjusting for smoking history, comorbidities, and emphysema severity. There was no significant difference in the plasma levels of the two RAGE ligands between cases and controls.

**Conclusions:**

sRAGE is significantly lower in patients with COPD than in age- and sex-matched individuals without airflow obstruction. Emphysema and chronic cor pulmonale are independent predictors of reduced sRAGE in COPD.

## Background

Chronic obstructive pulmonary disease (COPD) is a major cause of morbidity, disability, and mortality in industrialized countries [1]. It is characterized by an inflammatory response of the lung to inhaled noxious agents which brings about progressive airflow obstruction [1]. COPD also features a systemic inflammatory component with muscle wasting and weight loss [2,3].

The forced expiratory volume in one second (FEV_1_) is used in clinical practice for the diagnosis and staging of COPD, but it is deemed insufficient for the full characterization of patients with established COPD [4]. A large-scale prospective study is now underway to define clinically relevant COPD phenotypes, and identify biomarkers, correlated with such phenotypes, that might predict the disease progression and the effect of therapeutic interventions [5,6].

The receptor for advanced glycation end products (RAGE) is a 35 kD transmembrane receptor belonging to the immunoglobulin superfamily [7]. RAGE interacts with a variety of ligands including amyloid peptide, N-epsilon-carboxymethyl lysine (CML), S100 proteins, and the DNA-binding protein "high mobility group box 1" (HMGB1) [8]. Binding of RAGE with its ligands is thought to trigger a pro-inflammatory gene activation [9].

Soluble RAGE (sRAGE), an isoform of RAGE lacking transmembrane and cytosolic domains, acts as a decoy receptor for RAGE ligands in the extracellular compartment, and is believed to afford protection against inflammation and cell injury [10]. Reportedly, sRAGE levels are reduced in patients with coronary artery disease, rheumatoid arthritis, and idiopathic pulmonary fibrosis as compared with healthy subjects [11-14].

In a study comprising 61 patients with COPD and 42 healthy controls, Smith and coworkers showed that sRAGE is significantly correlated with FEV_1 _as percent predicted, the greater the degree of airflow obstruction the lower the plasma concentration of sRAGE [15].

Recently, Ferhani et al. [16] reported that RAGE is over-expressed in the airway epithelium and in the airway smooth muscle of smokers with COPD, and colocalizes with HMGB1. In that study, the circulating levels of sRAGE were not measured.

With the present study, we aimed at establishing whether plasma levels of sRAGE and of its ligands CML and S100A12 are correlated with the presence and severity of emphysema in a sample of 200 patients with COPD who were recruited into a multicenter European study on genetic susceptibility to the development of COPD. An equal sample of subjects without airflow obstruction served as controls.

As a secondary end-point, we looked for an association between sRAGE and chronic cor pulmonale in COPD.

## Methods

### Sample

The study sample included 200 patients with COPD and 201 controls who were part of a larger cohort enrolled in a case-control study aimed at assessing genetic susceptibility to the development of COPD [17-20].

The subjects, all white Caucasian, were evaluated at the outpatient clinic of the CNR Institute of Clinical Physiology, Pisa, Italy, between November 1, 2001 and September 30, 2003. Potential candidates were contacted through the family physicians in the city of Pisa.

Criteria for case recruitment were: (a) firm clinical diagnosis of stable COPD, (b) airflow obstruction as indicated by a post-bronchodilator ratio of forced expiratory volume in one second over forced vital capacity (FEV_1_/FVC) <0.7 and FEV_1 _≤70% of the predicted value [21], and (c) smoking history ≥20 pack-years.

Patients were excluded from the study if they had an established diagnosis of asthma, chronic lung disorders other than COPD or lung cancer, history of atopy, known alpha-1-antitrypsin deficiency, or a serum alpha-1-antitrypsin concentration <1.0 g/dL. Patients were also excluded if they had had a clinically confirmed acute exacerbation in the 4 weeks preceding the study entry.

By study design, the controls were recruited to match the COPD patients on age and gender. All the controls were current or former smokers with a smoking history ≥20 pack-years. Only individuals with no airflow obstruction were included in the control group (FEV_1_/FVC >0.7; FVC and FEV_1 _>80% of the predicted value). Individuals were excluded from the control group if they had a history of chronic lung disease or atopy, a family history of COPD, or had had an acute respiratory infection in the 4 weeks preceding the study entry.

### Study protocol

The protocol was approved by the local ethics committee (Comitato Etico, Azienda Ospedaliero-Universitaria Pisana, Pisa, Italy). Before entering the study, an informed written consent was obtained from all the subjects.

Detailed clinical history and physical examination were obtained in each participant. Definitions of comorbid conditions are reported in the online additional file. Lung function studies included the measurement of FVC and FEV_1 _(before and after bronchodilator), and of single breath diffusing capacity of the lung for carbon monoxide (DL_CO_). Spirometry, and DL_CO _measurements were performed in conformity with the ATS/ERS standards [22,23]. The severity of COPD was staged according to the GOLD guidelines [1].

Chronic cor pulmonale was rated present if there was evidence of persistent enlargement of the right ventricle on at least two consecutive echocardiographic studies obtained in the year preceding the study entry. Diagnostic criteria included an end-diastolic right ventricular diameter >26 mm in the parasternal long-axis view, or a ratio of right-to-left end-diastolic ventricular diameter >1 in the apical four-chamber view [24]. Right ventricle hypertrophy was rated present if the thickness of the right ventricular free wall was ≥ 7 mm in the subcostal view [24].

Postero-anterior and lateral digital chest radiographs were obtained in all the subjects at the time of enrollment in the study, and were examined by two chest physicians for the presence of heart and pulmonary abnormalities. The COPD patients were also invited to complete a self-administered quality-of-life questionnaire [25]. Upon inclusion, a blood sample (in lithium heparin) was obtained from all the subjects for genomic studies. Plasma aliquots were stored at -80°C until futher processing.

### Measurement of sRAGE

The plasma concentration of sRAGE was determined using a double-sandwich ELISA method (DuoSet ELISA kit, R&D Systems, Minneapolis, MN). The methodology is described in full elsewhere [26], and is reported briefly in the online additional file. With this assay, the lower limit of detection of sRAGE is 21.5 pg/mL [26]. Our ELISA method measures total sRAGE because it utilizes an antibody directed against the extracellular domain, so it cannot distinguish the shedded isoform from the splice variant of RAGE (also known as endogenous secretory RAGE, or esRAGE).

We also measured the plasma concentrations of two RAGE ligands, CML and S100A12, by ELISA method (see online additional file). CML can be generated on proteins by a myeloperoxidase-dependent pathway when neutrophils are activated [27]. Similarly, S100A12 is secreted by cytokine-activated neutrophils [28]. Since COPD is characterized by neutrophil activation, we thought it appropriate to measure the circulating levels of the two ligands in our study sample.

### Computed tomography

Computed tomography (CT) of the thorax was obtained in COPD patients within three months of their recruitment into the study. It was performed on a Toshiba Aquilion 64 detector row scanner (Toshiba, Japan) with the patient breath-holding at full inspiration for 10 seconds. Acquisition setting was 120 kVp with mAs modulated according to the patient's attenuation as assessed before scan acquisition (range, 60 to 250 mAs). Slice thickness was set at 0.65 mm. No contrast medium was infused. Scans were reconstructed in the axial, sagittal and coronal planes. Images were viewed using a window level of -600 Hounsfield Units (HU) and a width of 1,500 HU, and were examined independently by a chest radiologist and a chest physician for the presence of areas of low attenuation and vascular disruption. The two raters were blinded to clinical and lung function data.

Maximum intensity projection technique was used to evaluate vascular disruption, and minimum intensity projection to highlight focal areas of low attenuation in the lung parenchyma [29].

The severity of emphysema was scored on a nonparametric scale from 0 (no emphysema) to 100 using the panel-grading (PG) method of Thurlbeck et al. [30]. This consists of 16 inflation-fixed, paper-mounted, midsagittal whole lung sections that are arranged at intervals of 5 between 0 and 50, and at intervals of 10 between 60 and 100. A score of 5 or less is consistent with trace emphysema, a score of 10 to 30 indicates mild emphysema, a score >30 to 50 moderate emphysema, and a score >50 to 100 severe emphysema [30]. In scoring emphysema on CT, the two raters examined sagittal lung sections, and gave them the score of the standard most closely similar, or a score between two standards. Examples are shown in the online additional file. The PG scores by the two independent raters were averaged.

### Statistical analysis

Differences between and within groups were assessed by Fisher's exact test for the categorical variables, and by Mood's median test for the continuous variables. Continuous variables in the text and in the tables are reported as median and interquartile range (IQR). The scatter plot of the PG of emphysema by the two independent raters was tested for departure from perfect agreement by fitting a simple linear regression model and testing the null hypothesis that the intercept is equal to zero and the slope is equal to one, jointly.

We utilized the data from the 200 COPD patients and the 201 age and sex-matched controls to estimate the effects on the three quartiles (25^th^, 50^th^, and 75^th ^percentile) of sRAGE of the following variables: pack-years of smoking, coronary artery disease, diabetes mellitus, dyslipidemia, airflow obstruction as reflected by FEV_1_% predicted, and emphysema on CT. Airflow obstruction was dichotomized as absent (FEV_1_>80%) or present (FEV_1_<80%). Emphysema was categorized as absent, mild, moderate, or severe based on the PG score. "Absent" emphysema with no airflow obstruction was the reference category. We tested for trends across the varying degrees of severity of emphysema. We included chronic cor pulmonale in a secondary analysis. Sex and age were matching variables by design, and their effect could not be assessed. The potential dependence of the observations within each matched group was taken into account by using cluster bootstrap for the inference on the three quartiles of sRAGE. The statistical analysis was performed with Stata version 10 (StataCorp, College Station, TX).

## Results

### Sample characteristics

The baseline characteristics of the study sample are summarized in table 1. The control subjects were matched to the COPD patients on age and gender, and did not differ from them with regard to body mass index (BMI). The proportion of current smokers was nearly identical in the two groups, but the cumulative exposure to cigarette smoking was significantly higher in COPD than in controls. The two groups had a similar prevalence of comorbid conditions.

**Table 1:** Baseline characteristics of the study sample

Characteristics	COPD (n = 200)	No COPD (n = 201)	P-value
Age, years	66 (61-70)	65 (61-70)	0.258
Male sex	178 (89)	172 (86)	0.369
BMI (kg/m^2^)	27 (24-31)	28 (25-30)	0.508
Current smoker	97 (49)	101 (50)	0.766
Pack-years of smoking	48 (39-60)	40 (33-50)	<0.001
FEV_1_, % predicted	54 (42-65)	95 (88-105)	<0.001
DL_CO_, % predicted	76 (58-86)	96 (86-108)	<0.001
Chronic mucous hypersecretion	116 (58)	46 (23)	<0.001
Chronic cor pulmonale	47 (24)	0 (0)	<0.001
Emphysema	87 (44)	0 (0)	<0.001
			
*Comorbidity*			
Systemic arterial hypertension	95 (48)	75 (37)	0.043
Coronary artery disease	59 (30)	55 (27)	0.659
Heart failure	23 (12)	13 (6.5)	0.083
Dilated cardiomyopathy	6 (3)	6 (3)	1.000
Left heart valvular disease	8 (4)	5 (2.5)	0.416
Chronic atrial fibrillation	10 (5)	7 (3.5)	0.470
Prior stroke	2 (1)	3 (1.5)	1.000
Prior PE or DVT	5 (2.5)	3 (1.5)	0.503
Renal failure	0 (0)	0 (0)	1.000
Diabetes mellitus	22 (11)	30 (15)	0.298
Dyslipidemia	61 (31)	74 (37)	0.205
Thyroid dysfunction	18 (9)	11 (5.5)	0.183
Chronic hepatitis C	7 (3.5)	9 (4.5)	0.799
			
*Therapy*			
Inhaled bronchodilators	139 (70)	0 (0)	<0.001
Inhaled corticosteroids	128 (64)	0 (0)	<0.001
Oral theophylline	48 (24)	0 (0)	<0.001
Long-term oxygen	7 (3.5)	0 (0)	0.007
Cardiovascular drugs	122 (61)	108 (54)	0.158
Diuretics	52 (26)	30 (15)	0.006
Warfarin	15 (8)	10 (5)	0.311
Statins	37 (19)	47 (23)	0.269
Oral hypoglicemic drugs/insulin	17 (9)	21 (10)	0.609
Thyroid replacement therapy	7 (3.5)	4 (2)	0.380

Data are reported as median (interquartile range), or number (%).

Definitions of abbreviations: BMI = body mass index; FEV_1 _= forced expiratory volume in one second; DL_CO _= diffusing capacity of the lung for carbon monoxide; PE = pulmonary embolism; DVT = deep vein thrombosis.

Emphysema was consistently diagnosed by the two independent raters in 87 (44%) of 200 patients with COPD (see online additional file for inter-rater agreement). In the 87 emphysemic patients, the median PG score was 46 (IQR, 39 to 59). In the whole COPD sample, the PG score of emphysema was significantly correlated with FEV_1 _as % predicted (r = -0.62, p < 0.001), and with DL_CO _as % predicted (r = -0.67, p < 0.001).

Based on the PG score, the COPD sample was divided in three categories: no or mild emphysema (PG ≤ 30, n = 119), moderate emphysema (PG > 30 to 50, n = 56), and severe emphysema (PG > 50, n = 25).

As shown in table 2, the COPD patients with moderate to severe emphysema (PG > 30) had significantly lower BMI, FEV_1 _and DL_CO_, and featured a significantly higher prevalence of chronic cor pulmonale and a worse quality of life than those with no or mild emphysema (PG ≤ 30).

**Table 2:** Baseline characteristics of COPD patients in relation to the presence and severity of emphysema

Characteristics	No or mild (n = 119)	Moderate to severe (n = 81)	P-value
Age, years	66 (60-69)	67 (62-71)	0.475
Male sex	105 (88)	73 (90)	0.819
BMI (kg/m^2^)	29 (26-31)	25 (23-27)	<0.001
Current smoker	60 (50)	37 (46)	0.565
Pack-years of smoking	46 (38-59)	50 (40-60)	0.152
FEV_1_, % predicted	60 (51-66)	42 (32-53)	<0.001
DL_CO_, % predicted	83 (74-97)	57 (47-71)	<0.001
Chronic mucous hypersecretion	65 (55)	51 (63)	0.248
Chronic cor pulmonale	16 (13)	31 (38)	<0.001
QoL questionnaire, total score	28 (19-41)	40 (21-60)	0.009
			
*Comorbidity*			
Systemic arterial hypertension	67 (56)	28 (35)	0.004
Coronary artery disease	37 (31)	22 (27)	0.636
Heart failure	12 (10)	11 (14)	0.502
Diabetes mellitus	15 (13)	7 (9)	0.492
Dyslipidemia	44 (37)	17 (21)	0.019
			
*Therapy*			
Inhaled bronchodilators	74 (62)	65 (80)	0.008
Inhaled corticosteroids	71 (60)	57 (70)	0.135
Oral theophylline	22 (18)	26 (32)	0.030
Cardiovascular drugs	80 (67)	42 (52)	0.039
Diuretics	28 (24)	24 (30)	0.412
Statins	25 (21)	12 (15)	0.354

Data are reported as median (interquartile range), or number (%).

Definitions of abbreviations: QoL = quality of life.

For the other abbreviations see table 1.

Systemic arterial hypertension and dyslipidemia prevailed significantly in the patients with PG ≤ 30 with respect to those having PG > 30 (table 2). In the latter group, significantly more patients were receiving inhaled bronchodilators and oral theophylline than those with PG ≤ 30 (table 2).

### Circulating levels of sRAGE

The median circulating level of sRAGE in COPD patients was 652 pg/mL (IQR, 484 to 1076 pg/mL), and was significantly lower (p = 0.007) than in controls (median 869 pg/mL, IQR 601 to 1240 pg/mL).

Among the COPD patients, there was no significant difference in the levels of sRAGE between current and former smokers, nor there was any difference in relation to the cumulative smoking history (table 3).

**Table 3:** sRAGE in 200 COPD patients (univariate analysis)

Characteristics	n	Median	IQR	P-value
*Smoking habits*				
Current smoker	97	677	483-1076	0.777
Former smoker	103	638	492-1063	
*Pack-years of smoking*				
>48	96	628	449-1055	0.479
≤48	104	669	484-1076	
*FEV*_*1*_*, % predicted*				
≥50	122	660	503-1078	0.015
<50	62	763	538-1138	
<30	16	435	247-544	
*DL*_*CO*_*, % predicted*				
>76	96	745	546-1051	0.007
≤76	104	612	428-1076	
*Emphysema*				
No or mild	119	715	532-1174	0.003
Moderate	56	644	482-1041	
Severe	25	465	243-644	
*Chronic cor pulmonale*				
yes	47	534	321-741	0.002
no	153	715	529-1164	
*Systemic arterial hypertension*				
yes	95	658	484-1064	0.999
no	105	644	477-1076	
*Coronary artery disease*				
yes	59	681	560-1040	0.352
no	141	631	465-1087	
*Heart failure*				
yes	23	886	583-1252	0.376
no	177	641	465-1039	
*Diabetes mellitus*				
yes	22	920	735-1123	0.003
no	178	630	475-1069	
*Dyslipidemia*				
yes	61	681	508-1078	0.539
no	139	631	480-1062	
*Inhaled corticosteroids*				
yes	128	642	482-1076	0.880
no	72	671	484-1078	
*Statins*				
yes	37	681	558-1033	0.713
no	163	641	470-1082	

Definitions of abbreviations: sRAGE = soluble receptor for advanced glycation end products (pg/mL); IQr = interquartile range. For the other abbreviations see table 1. The cutpoints of FEV_1 _% predicted are based on GOLD stage (see ref. [1]). The cutpoints for pack-years of smoking and DL_CO _% predicted are the median value in the whole sample.

With regard to lung function, there was a significant relationship between sRAGE and the degree of airflow obstruction, the lower the level of sRAGE the greater the airflow obstruction (table 3). Similarly, sRAGE was significantly lower in the COPD patients with DL_CO _below the median value than in those with DL_CO _above the median, so indicating a relationship with functional emphysema (table 3).

**Figure 1:**
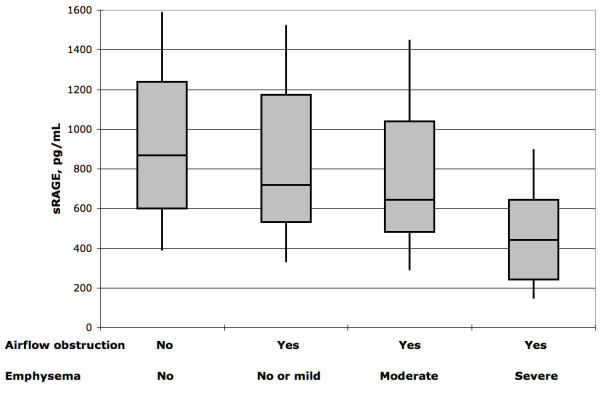
**Box-and-whisker plot of the plasma concentration of soluble receptor for advance glycation end products (sRAGE) in the study sample (n = 401)**. No airflow obstruction, no emphysema (n = 201). Airflow obstruction, no or mild emphysema (n = 119). Airflow obstruction, moderate emphysema (n = 56). Airflow obstruction, severe emphysema (n = 25). Line in box: median. Box height: interquartile range. Whiskers: 10^th ^and 90^th ^percentile. P < 0.001 by Mood's median test.

A significant difference was confirmed when the comparison was made with the severity of structural emphysema on CT, sRAGE being the lowest in the patients with severe emphysema (table 3, figure 1). Also, sRAGE was significantly lower in the COPD patients with chronic cor pulmonale than in those without (table 3).

Diabetes was associated with significantly higher values of sRAGE, whereas cardiovascular disorders, dyslipidemia, and use of inhaled corticosteroids or statins had no effect on the plasma concentration of sRAGE (table 3).

Among the controls, there was no significant difference in the circulating levels of sRAGE in relation to smoking habits, relevant comorbidities, or statin use (table 4).

**Table 4:** sRAGE in 201 controls (univariate analysis)

Characteristics	n	Median	IQR	P-value
*Smoking habits*				
Current smoker	101	884	622-1284	0.888
Former smoker	100	833	579-1035	
*Pack-years of smoking*				
>40	101	869	601-1305	1.000
≤40	100	856	600-1203	
*Systemic arterial hypertension*				
yes	75	790	619-1207	0.382
no	126	899	591-1248	
*Coronary artery disease*				
yes	55	874	671-1055	1.000
no	146	855	570-1292	
*Heart failure*				
yes	13	772	663-948	0.568
no	188	876	598-1247	
*Diabetes mellitus*				
yes	30	907	680-1124	0.435
no	171	851	584-1247	
*Dyslipidemia*				
yes	74	853	621-1008	0.884
no	127	874	580-1291	
*Statins*				
yes	47	772	617-951	0.182
no	154	899	582-1295	

Definitions of abbreviations: sRAGE = soluble receptor for advanced glycation end products (pg/mL); IQr = interquartile range. The cutpoint of 40 pack-years is the median value measured in the whole sample.

**Figure 2:**
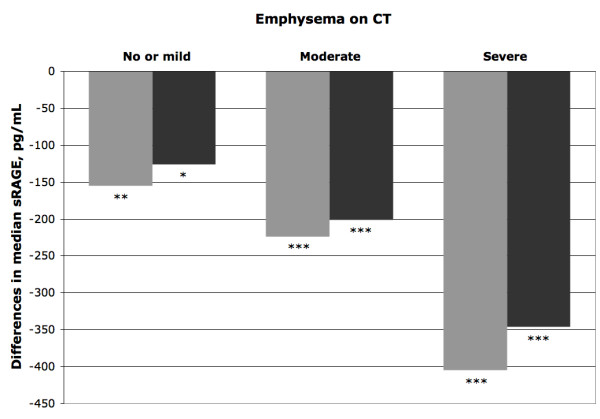
**Differences in median sRAGE between COPD patients (n = 200) and control subjects (no airflow obstruction and no emphysema, n = 201) taken as the referent category**. COPD patients are categorized as a function of the presence and severity of emphysema on computed tomography (CT). Grey bars: unadjusted difference. Black bars: difference adjusted for airflow obstruction, pack-years of smoking, coronary artery disease, diabetes, and dyslipidemia. * p < 0.05, ** p < 0.01, *** p < 0.001 against the referent category.

Figure 2 shows the differences in median sRAGE between the COPD patients, categorized as a function of emphysema severity, and the controls taken as the reference category. The difference in median sRAGE increased with increasing emphysema severity, and was statistically significant at all levels of severity with respect to the reference category. The observed differences remained statistically significant even after adjusting for other independent variables such as airflow obstruction, pack-years of smoking, coronary artery disease, diabetes, and dyslipidemia.

The unadjusted difference in median sRAGE between the COPD patients with and those without chronic cor pulmonale was -181 pg/mL (table 3). After adjusting for smoking history, comorbid conditions, and emphysema severity, the difference in median sRAGE between the two groups decreased to -168 pg/mL, but remained statistically significant (p < 0.001).

The interaction between emphysema and chronic cor pulmonale was not statistically significant. This means that both variables are important predictors of reduced sRAGE, but the effect of either variable does not vary in relation to the presence or absence of the other.

### Circulating levels of N-epsilon-carboxymethyl lysine and S100A12

The median circulating level of CML in COPD patients was 53 mcg/mL (IQR, 35 to 72 mcg/mL), and did not differ (p = 0.42) from that of controls (median 50 mcg/mL, IQR 35 to 72 mcg/mL). Similarly, we found no difference in the plasma concentration of S100A12 between cases and controls, the median value of S100A12 being 42 ng/mL (IQR, 30 to 61 ng/mL) in the COPD patients, and 42 ng/mL (IQR, 33 to 57 ng/mL) in the controls (p = 0.71).

Among the patients with COPD, there was no significant correlation between sRAGE and CML (r = -0.062, p = 0.38), or S100A12 levels (r = -0.048, p = 0.50). Lack of correlation between sRAGE and the two RAGE ligands was confirmed in the controls (r = 0.007, p = 0.92 vs CML; r = 0.013, p = 0.86 vs S100A12).

## Discussion

Over recent years, a number of reports showed that, relative to healthy controls, sRAGE is significantly reduced in a variety of disorders including coronary artery disease, rheumatoid arthritis, and idiopathic pulmonary fibrosis [11-14]. Since sRAGE acts as a decoy receptor for RAGE ligands, reduced levels of sRAGE are thought to be expression of an impaired immunologic control [11-14].

In the study by Smith and coworkers, circulating sRAGE was significantly correlated with FEV_1 _as percent predicted, the greater the degree of airflow obstruction the lower the plasma concentration of sRAGE [15]. In a subset of 36 COPD patients, no significant relationship was observed between the plasma concentration of sRAGE and the lung diffusing capacity as reflected by DL_CO _[15]. In that study, radiologic imaging of the chest was not available, so the relationship between sRAGE and emphysema could not be assessed.

In the present study, we found that circulating sRAGE is significantly lower in patients with stable COPD than in subjects without airflow obstruction who are matched to COPD patients on age and gender, and who also feature very similar comorbid conditions.

The reduction of sRAGE in COPD was strongly associated with the impairment of lung diffusing capacity, and the severity of structural emphysema as seen on CT. The association of sRAGE with emphysema remained statistically significant after adjusting for a number of independent variables including cumulative smoking history, coexistence of coronary artery disease, diabetes mellitus, or dyslipidemia.

In our sample, there were no cases of interstitial lung diseases since, by design, all the patients with chronic lung disorders other than COPD were excluded from the study.

One subject only was affected by rheumatoid arthritis -- a 64-year old male who had normal lung function (FEV_1_/FVC >0.7, FEV_1 _92% predicted, and DL_CO _100% predicted), and no abnormality on chest radiography. In this subject, the plasma concentration of sRAGE was 292 pg/mL.

It appears, therefore, that emphysema is independently associated with the level of sRAGE in patients with COPD.

In contrast to other tissues, membrane-bound RAGE is highly expressed in the normal adult human lung, especially in the alveolar epithelial cells [31-33]. RAGE is thought to have a homeostatic function in the lung for it enhances the adherence of type I epithelial cells to the extracellular matrix [32], and is implicated in the differentiation of type II into type I epithelial cells, a crucial step in the process of alveolar repair [34].

So, the reduced levels of sRAGE we observed in the patients with moderate to severe emphysema could be the consequence of the extensive disruption of alveoli and alveolar walls that is the hallmark of emphysema.

Alternatively, the reduced levels of sRAGE in emphysemic patients may reflect the exposure to a high burden of RAGE ligands. This could, in turn, be caused by the release of pro-inflammatory cytokines and inflammatory mediators that is known to occur in COPD [35].

We found no significant difference, between cases and controls, in the plasma concentrations of the RAGE ligands CML and S100A12. This is at variance with the results of three recent studies showing that: (a) CML is detected in the epithelial lining fluid from peripheral airways in patients with COPD, and correlates with the severity of airflow obstruction [36]; (b) S100A12 concentration in the sputum of patients with COPD is significantly higher than in healthy subjects [37]; (c) HMGB1 levels in induced sputum are significantly higher in asthmatic patients than in controls, and correlate significantly with the severity of asthma and the percent of neutrophils in sputum [38].

These data suggest compartmentalization of RAGE ligands in the airway lumen in patients with obstructive lung diseases, and may explain why we did not find any significant increase in the circulating levels of CML and S100A12 in our COPD sample.

Since RAGE is considered a marker of alveolar epithelial cell integrity, it may be speculated that disruption of alveolar integrity is associated with downregulation of RAGE. This hypothesis was tested in animal models recapitulating idiopathic pulmonary fibrosis (IPF), and in lung specimens from patients with known IPF [13,14]. These studies indicate that: (a) RAGE is downregulated in murine models of IPF; (b) RAGE-null mice are prone to develop fibrotic lesions in their lungs; (c) in humans, RAGE and sRAGE transcripts are significantly reduced in lPF lungs as compared with normal lungs. Taken together these findings support the concept that RAGE may have a protective role in the lungs, and that loss of RAGE contributes to IPF pathogenesis [13,14].

By contrast, immunohistochemical studies show that RAGE is over-expressed in the conducting airways [16] and alveolar walls [39] of patients with COPD. Recently, a proteomic screening study of the lung tissue in patients with IPF and with COPD revealed that full length-RAGE is reduced in both diseases whereas esRAGE levels decline in IPF but not in COPD [40]. So, it may be that specific RAGE variants are involved in COPD. This issue warrants further investigation.

Although it was not the primary objective of our study, we found that chronic cor pulmonale is strongly and independently associated with reduced levels of sRAGE in COPD.

Chronic cor pulmonale may develop in COPD as a consequence of anatomic remodeling of the pulmonary vasculature, and sustained vasoconstriction due to chronic hypoxia and superimposed respiratory acidosis [41].

Recent experimental data suggest that reactive oxygen species, released during inflammation, may impact on the structure and function of the right ventricle [42]. So, the low concentrations of sRAGE we measured in the patients with chronic cor pulmonale could again be regarded as indicating exposure to high levels of RAGE ligands. This hypothesis should be further tested in patients with established pulmonary arterial hypertension.

We acknowledge that our study has some limitations. First, given the cross-sectional nature of the study, we obtained a single determination of sRAGE, FEV_1 _and DL_CO_, and a single CT scan study. This precluded the possibility of evaluating whether changes in sRAGE over time are predictive of a decline in lung function, or worsening of emphysema in patients with established COPD. Second, we measured total circulating sRAGE because the ELISA method we used does not differentiate between the shedded isoform and that generated by alternative splicing (esRAGE). Third, we did not measure the expression of RAGE in the lung tissue and, therefore, we could not assess the relationship between the membrane-bound and the soluble isoforms of the receptor.

Investigating on the dynamics of RAGE and its soluble isoforms in COPD seems warranted in view of the results of two recent meta-analyses of genome-wide, population-based studies [43,44]. A strong association was found between lung function (as reflected by the FEV_1_/FVC ratio) and single nucleotide polymorphisms in the AGER gene encoding RAGE, which is a plausible candidate for causal association [43,44].

## Conclusions

In summary, we found that circulating sRAGE is significantly reduced in COPD patients with respect to age- and sex-matched controls. Emphysema and chronic cor pulmonale are independent predictors of reduced sRAGE levels in COPD.

## Competing interests

The authors declare that they have no competing interests.

## Authors' contributions

MM designed the study; MM, SM, and EF contributed to acquisition and interpretation of data; GB and FC measured sRAGE; MB contributed to statistical analysis; MM and MB drafted the manuscript. All the authors read and approved the final version of the manuscript.
